# Multiple congenital malformations in a litter of feline fetuses associated with gestational exposure to a synthetic estrus-suppressing progestin

**DOI:** 10.1186/s12917-026-05506-8

**Published:** 2026-04-28

**Authors:** Wellida Karinne Lacerda, Ana Letícia Pereira Fernandes, Mônica Shinneider de Sousa, Pedro Lucas Chaves de Gusmão, Lucas Rannier Ribeiro Antonino Carvalho, Ricardo Barbosa Lucena

**Affiliations:** 1 Postgraduate Program in Animal Science, Universidade Federal da Paraiba, Areia, PB 58397-000 Brazil; 2 PatoBiology Laboratory, Universidade Federal da Paraiba, Areia, PB 58397-000 Brazil; 3 Department of Physiology and Pharmacology, Karolinska Institutet, Solnavägen 9, Stockholm, S-17177 Sweden

**Keywords:** Congenital malformations, Developmental abnormalities, Estrus suppression, Feline, Reproductive toxicity, Synthetic progestins

## Abstract

**Background:**

Congenital malformations in domestic cats are traditionally considered uncommon; however, recent evidence suggests they may represent an underrecognized cause of neonatal mortality. Most reported anomalies occur as isolated defects in individual animals, while the occurrence of multiple severe malformations within the same fetus or affecting an entire litter appears to be rare. Exposure to exogenous substances during critical windows of embryonic development has been proposed as a potential contributing factor. Reports describing congenital abnormalities in feline fetuses following gestational exposure to synthetic progestins remain scarce.

**Case presentation:**

A three-year-old intact domestic shorthair cat was presented for veterinary evaluation due to dystocia approximately 65 days after repeated escape episodes from the household. In an attempt to suppress estrus and prevent mating, the owner administered two subcutaneous injections of a commercially available estrus-suppressing agent containing medroxyprogesterone acetate, before pregnancy was clinically recognized. Cesarean section revealed three dead fetuses, all presenting multiple congenital malformations. Cleft palate and arthrogryposis were observed in all fetuses, with arthrogryposis limited to the thoracic limbs in two and generalized in the third. Two female fetuses exhibited clitoromegaly consistent with virilization of the external genitalia. Additional findings included markedly reduced lung size suggestive of pulmonary hypoplasia in one fetus, cranium bifidum associated with meningocele in another, and severe axial and ventral body wall defects compatible with schistosomus retroflexus in the third. Rapid tests for feline immunodeficiency virus, feline leukemia virus, and feline panleukopenia virus in the queen were negative. Histopathological examination revealed diffuse vascular congestion and moderate autolysis, consistent with intrauterine fetal death, without evidence of inflammatory or infectious disease.

**Conclusions:**

Although a direct causal relationship cannot be established, the temporal sequence of events and the spectrum of malformations observed are suggestive of disruption of early embryonic development. This case highlights the potential risks associated with the use of synthetic estrus-suppressing progestins in intact queens when pregnancy status is unknown. Increased awareness, cautious use of hormonal contraceptives, and further well-documented reports are warranted to better elucidate their potential impact on fetal development in cats.

## Background

Congenital malformations in domestic cats have traditionally been considered relatively uncommon [1–5] when compared to other domestic species [6]. However, recent studies indicate that congenital anomalies may represent an important and underrecognized cause of neonatal mortality in kittens, accounting for a substantial proportion of early-life deaths [7]. Available reports describe a wide variety of congenital defects affecting different organ systems, including the craniofacial region, musculoskeletal system, nervous system [8], and cardiovascular system [9], which are most often reported as isolated findings in individual animals. In contrast, the occurrence of multiple severe congenital malformations within the same individual, and particularly affecting all fetuses within a single litter, appears to be rare. The etiology of congenital abnormalities in cats is frequently multifactorial and may involve genetic factors, infectious agents, nutritional imbalances, and exposure to exogenous substances during critical periods of embryonic development [1, 7, 10].

Among potential exogenous factors, hormonal manipulation of reproduction has historically been practiced in small animal medicine, particularly for estrus suppression in intact females [11]. However, unlike natural progesterone, synthetic progestins may exhibit androgenic and other endocrine-disrupting properties, and their use has been associated with a variety of adverse effects in domestic animals [11]. Of particular concern is gestational exposure to these compounds, which has been suggested to interfere with normal fetal development [12], especially when administration occurs during early pregnancy, often before gestation is clinically recognized [13].

Despite a gradual decline in their use, estrus-suppressing progestins remain accessible and are still administered in some settings, frequently without confirmation of pregnancy status. Reports describing congenital malformations in feline fetuses following gestational exposure to these agents remain limited, and detailed pathological characterization of such cases is scarce. Therefore, the objective of this report is to describe the clinical history and pathological findings observed in a litter of three feline fetuses presenting multiple congenital malformations following gestational exposure to a synthetic estrus-suppressing progestin, and to discuss the findings in the context of embryological development and the existing literature.

## Case report

A three-year-old intact domestic shorthair cat (*Felis catus*), domiciled and with no relevant previous medical history, was presented for veterinary evaluation due to signs of abdominal pain and restlessness. Although domiciled, the queen had a free-roaming lifestyle, with unrestricted access to the outdoor areas of the house and also to the street, which limited close day-to-day monitoring. According to the owner, the cat initially escaped from the household for approximately five days and returned apparently healthy. One week later, a second escape episode occurred, with the animal returning on the same day.

In an attempt to prevent estrus and potential mating during these episodes, the owner administered (1.0 mL administered subcutaneously) a commercially available estrus-suppressing injectable formulation, containing medroxyprogesterone acetate as the active ingredient. Approximately fifteen days after the first administration, the cat escaped again and received a second subcutaneous injection of the same estrus-suppressing agent. At the time of administration, pregnancy had not been clinically recognized.

Approximately 65 days after the first escape episode, the cat developed restlessness and signs of abdominal pain and was taken for veterinary evaluation. Clinical examination revealed advanced-term pregnancy associated with restlessness, abdominal pain, and vaginal discharge, findings consistent with dystocia. Ultrasonography confirmed advanced gestation and showed absence of detectable fetal cardiac activity in all fetuses, consistent with fetal death prior to cesarean section. A cesarean section was therefore performed. At the time of surgery, the mammary glands appeared to be within the expected range for a pregnant queen. However, the owner was unable to provide reliable information regarding mammary development during gestation, and subtle changes may have gone unnoticed because of the animal’s free-roaming lifestyle. During surgery, three fetuses were identified, all of which were dead and exhibited multiple congenital malformations. Rapid point-of-care tests for feline immunodeficiency virus (FIV) and feline leukemia virus (FeLV) (SNAP FIV/FeLV Combo, IDEXX Laboratories, Inc., USA) and for feline panleukopenia virus (FPV Ag ECO Vet, ECO Diagnóstica, Brazil) were negative. The same three fetuses identified during surgery were subsequently submitted for complete necropsy.

Gross examination of the litter revealed multiple congenital anomalies with both shared and individual features (Table 1). Two fetuses were female, and one was male; both female fetuses exhibited clitoromegaly consistent with virilization of the external genitalia.

**Table 1:** Multiple congenital malformations in three feline fetuses from a single litter

Fetus	Sex	Malformations observed
Fetus 1	Female	Cleft palate; arthrogryposis (thoracic limbs); clitoromegaly.
Fetus 2	Female	Cleft palate; arthrogryposis (all four limbs); clitoromegaly; cranium bifidum with meningocle, markedly reduced lung size.
Fetus 3	Male	Cleft palate; arthrogryposis (all four limbs); pronounced scoliosis and lordosis; large ventral body wall defect with evisceration (schistosomus retroflexus); mandibular brachygnathia; cranial flattening; reduced ossification of the calvarium; markedly reduced lung size.

**Fig. 1:**
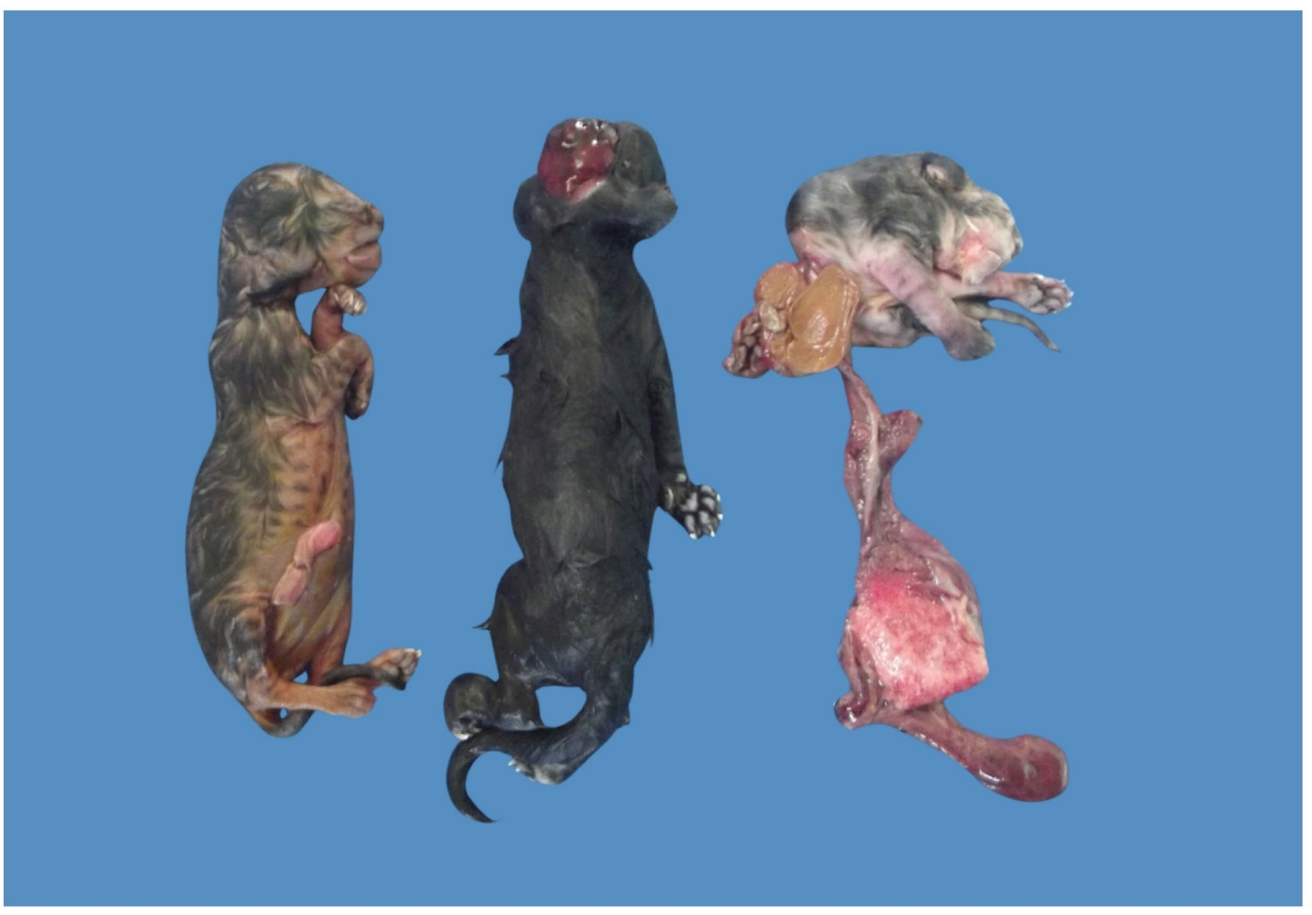
External appearance of three feline fetuses from the same litter presenting multiple congenital malformations. Fetuses are identified from left to right as 1, 2, and 3. All fetuses exhibited arthrogryposis. Fetus 1 showed limb contractures predominantly affecting the thoracic limbs. Fetus 2 presented cranium bifidum associated with meningocele and arthrogryposis affecting all four limbs. Fetus 3 exhibited severe axial deformities with generalized arthrogryposis and a large ventral body wall defect with visceral exteriorization, consistent with schistosomus reflexus

On external examination, arthrogryposis was present in all three fetuses (Fig. 1). In Fetus 1, joint contractures were restricted to the thoracic limbs. In Fetus 2, arthrogryposis affected all four limbs. In Fetus 3, limb contractures were generalized and more severe, markedly affecting all four limbs.

Marked external cranial abnormalities were observed in Fetuses 2 and 3. Fetus 2 exhibited a large cranial vault defect characterized by failure of calvarial bone closure and absence of overlying skin, with a protruding sac-like structure consistent with cranium bifidum associated with meningocele. Fetus 3 showed severe craniofacial and axial deformities, including mandibular brachygnathia, cranial flattening, and reduced ossification of the calvarial bones. This fetus also presented pronounced vertebral curvature abnormalities, including scoliosis and lordosis, together with a large ventral body wall defect with exteriorization of abdominal viscera, findings consistent with schistosomus reflexus.

**Fig. 2:**
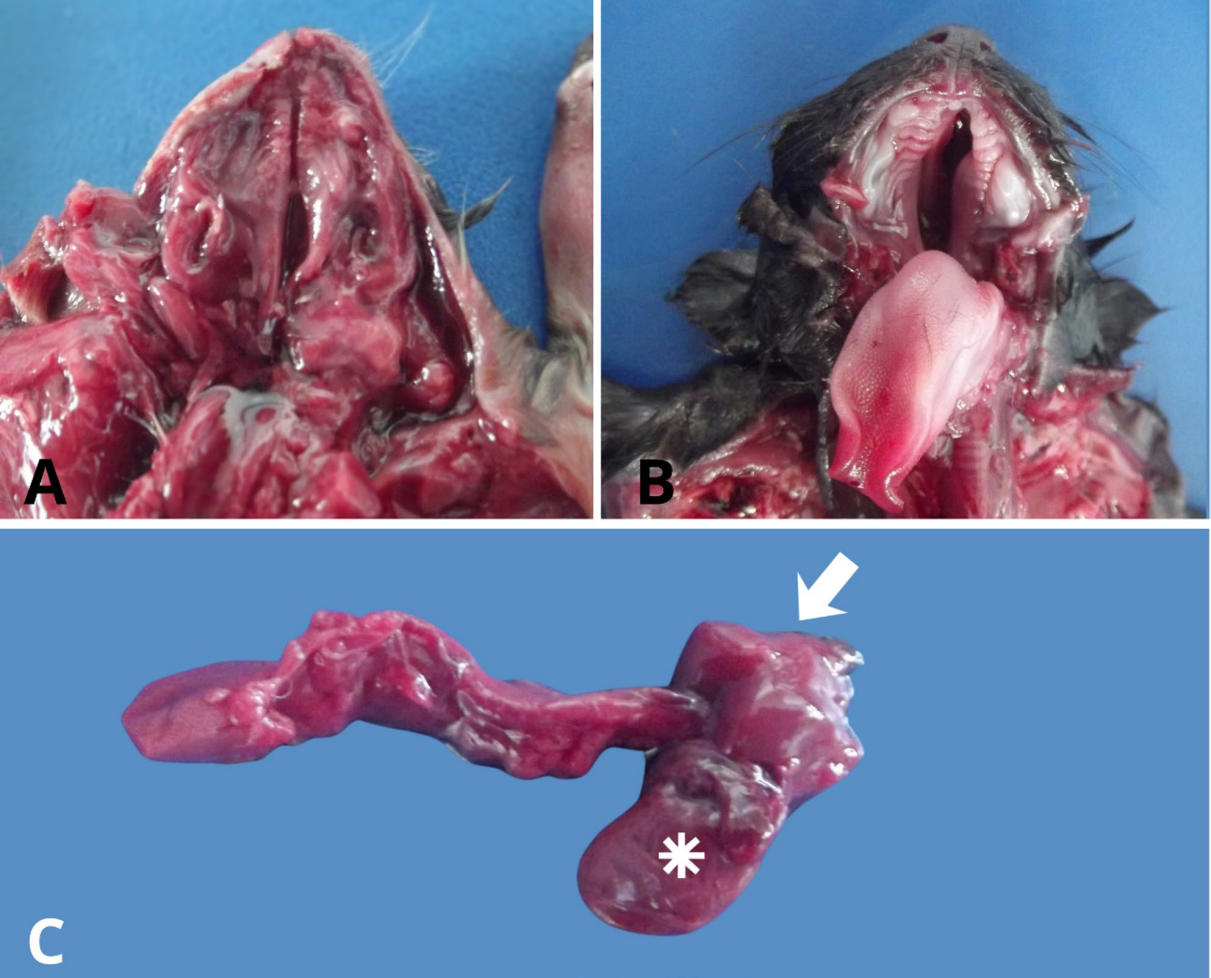
Oral and thoracic findings in two fetuses from the affected litter. **A** Complete cleft palate in Fetus (1) (**B**) Complete cleft palate in Fetus (2) (**C**) Pulmonary hypoplasia in Fetus 2 (arrow), with markedly reduced lung volume compared to the relative size of the heart (asterisk)

Upon opening of the oral cavity, complete cleft palate was identified in all three fetuses (Fig. 2A–B). Internal examination revealed marked pulmonary hypoplasia in Fetuses 2 and 3 (Fig. 2C). The uterus in Fetuses 1 and 2 was small but grossly unremarkable, a finding considered compatible with fetal immaturity and stillbirth. No additional internal structural abnormalities were observed beyond those externally evident.

Tissue samples from major internal organs, central nervous system, femur, and ribs were collected and fixed in 10% buffered formalin. Soft tissues were routinely processed, embedded in paraffin, sectioned at 4 μm, and stained with hematoxylin and eosin, while bone samples were decalcified in acid solution prior to processing. Histopathological examination revealed diffuse vascular congestion and a moderate degree of autolysis across examined tissues, consistent with intrauterine fetal death, without evidence of inflammatory, infectious, or additional structural lesions beyond those identified macroscopically.

According to follow-up information obtained from the owner, the queen did not subsequently develop fibroadenomatous hyperplasia or malignant mammary neoplasia.

## Discussion

The present case describes a constellation of congenital malformations affecting three feline fetuses, characterized by craniofacial, musculoskeletal, neural, and ventral body wall defects, observed following documented gestational exposure to a synthetic estrus-suppressing progestin. The heterogeneity and severity of the anomalies, coupled with their occurrence within the same gestation, suggest a significant developmental disturbance occurring during early embryogenesis [14, 15]. Although congenital malformations in cats are relatively uncommon, the pattern observed in this case aligns with defects known to arise during critical windows of organogenesis, when embryonic tissues are highly susceptible to exogenous insults [14–16].

Cleft palate was identified in all three fetuses, indicating a shared disruption of craniofacial development. In felines, palatal formation occurs during mid-organogenesis and relies on the precise growth, elevation, and subsequent fusion of the palatal shelves. Disruption of any of these tightly regulated processes may result in palatoschisis, a congenital defect that has been frequently associated with teratogenic exposure during critical periods of embryonic development [15]. The consistent presence of cleft palate across all fetuses supports the hypothesis of a common developmental insult occurring within a narrow embryological timeframe. In addition, arthrogryposis was observed in all individuals, albeit with varying degrees of severity. In two fetuses, joint contractures were limited to the thoracic limbs, whereas in the third fetus arthrogryposis was generalized and accompanied by severe axial malformations. This gradient of musculoskeletal involvement suggests a time- and severity-dependent developmental insult, consistent with early disruption of neuromuscular development and reduced fetal movement during critical stages of limb and spinal formation [17].

This combination of cleft palate and arthrogryposis has also been recognized in other developmental settings. In humans, arthrogryposis multiplex congenita is a phenotypically and genetically heterogeneous condition, and cleft palate may occur as part of its broader developmental spectrum [18]. Likewise, cleft palate is a multifactorial defect influenced by both genetic and environmental factors [19], and folate-related disturbances have been discussed as contributors to at least a subset of craniofacial malformations [20], including those linked to antifolate exposure [21]. In veterinary medicine, an arthrogryposis–palatoschisis complex has been described in small ruminants, particularly in inherited syndromes and in teratogenic plant-associated malformations [6]. In the present case, however, no clinical history suggested antifolate exposure or another clearly defined syndromic background, while the concurrent clitoromegaly in both female fetuses adds support to an endocrine-mediated developmental disturbance. Thus, although alternative causes cannot be entirely excluded, the findings are overall more suggestive of an association with gestational synthetic progestin exposure.

The identification of cranium bifidum associated with meningocele in one fetus further supports involvement of early neurulation processes. Neural tube defects arise from failure of normal neural tube closure during early embryogenesis, a process that precedes and directly influences cranial ossification and proper formation of the calvarium [14]. Although such defects are not classically associated with synthetic progestins, their presence in this case may reflect a broader disruption of embryonic development rather than a single-target effect.

Similarly, although schistosomus reflexus has historically been regarded as hereditary, emerging evidence suggests etiologic heterogeneity and possible environmental contributions [22]. In olive ridley sea turtles, the condition has been associated with increased mercury exposure and altered DNA methylation, supporting a role for gene–environment interactions [23]. In the present feline case, the association of schistosomus retroflexus with multiple congenital anomalies following gestational exposure to an endocrine-active compound is consistent with a broad early developmental disturbance, while causality cannot be established.

Of particular relevance in this case is the presence of clitoromegaly in both female fetuses, consistent with virilization of the external genitalia. Virilization of genetically female fetuses has been repeatedly described in association with exposure to synthetic progestins possessing androgenic activity [24]. Unlike natural progesterone, some synthetic progestins possess intrinsic androgenic activity or may alter the intrauterine endocrine milieu, potentially influencing pathways involved in fetal sexual differentiation [25, 26]. The observation that both female fetuses exhibited clitoromegaly, while the male fetus did not show comparable genital abnormalities, supports a sex-dependent effect and aligns with previously reported endocrine-disrupting outcomes of synthetic progestin exposure during gestation.

The clinical history revealed repeated administration of an estrus-suppressing progestin during a period compatible with early pregnancy, likely before gestation was recognized. The estimated timing of exposure overlaps with critical phases of organogenesis in the feline embryo, including neurulation, craniofacial development, musculoskeletal differentiation, and sexual differentiation [27]. This temporal association provides biological plausibility for the observed malformations, although causality cannot be definitively established based on a single case. Importantly, infectious causes commonly associated with congenital anomalies in cats [28] were excluded through negative testing for feline immunodeficiency virus, feline leukemia virus, and feline panleukopenia virus, further supporting a non-infectious etiology.

Histopathological examination revealed only diffuse congestion and moderate autolysis, findings consistent with intrauterine fetal death, and did not identify inflammatory or infectious lesions. The absence of specific histological alterations beyond structural malformations is not unexpected in cases of developmental anomalies arising from early embryonic disruption, particularly when fetal demise occurs prior to parturition [1]. As such, the diagnosis in this case relied primarily on gross pathological findings correlated with embryological timing and clinical history.

## Conclusion

This case report documents multiple severe congenital malformations in feline fetuses following gestational exposure to a synthetic estrus-suppressing progestin, highlighting a heterogeneous pattern of developmental abnormalities consistent with disruption during early organogenesis. The combination of craniofacial, musculoskeletal, neural, and genital anomalies underscores the potential risks associated with progestin administration in intact queens when pregnancy status is unknown. Although a direct causal relationship cannot be established, the findings emphasize the importance of cautious use of estrus-suppressing agents and contribute to the limited body of literature describing adverse developmental outcomes associated with gestational exposure to synthetic progestins in cats.

## Data Availability

The data supporting the findings of this study are included within the article. Additional information may be made available from the corresponding author upon reasonable request.
